# Ferroelectric order driven Eu^3+^ photoluminescence in BaZr_x_Ti_1−x_O_3_ perovskite

**DOI:** 10.1038/s41598-019-42897-1

**Published:** 2019-04-23

**Authors:** Giovanna Canu, Gregorio Bottaro, Maria Teresa Buscaglia, Chiara Costa, Oana Condurache, Lavinia Curecheriu, Liliana Mitoseriu, Vincenzo Buscaglia, Lidia Armelao

**Affiliations:** 1 ICMATE-CNR, Via De Marini 6, 16149 Genoa, Italy; 2 ICMATE-CNR and INSTM, Department of Chemical Sciences, University of Padua, Via F. Marzolo 1, 35131 Padua, Italy; 3 Department of Physics, Alexandru Ioan Cuza University, 11 Blvd. Carol I, 700506 Iasi, Romania; 4 Department of Chemical Sciences, University of Padua, Via F. Marzolo 1, 35131 Padua, Italy

**Keywords:** Electronic devices, Electronic materials

## Abstract

The ability to tune and enhance the properties of luminescent materials is essential for enlarging their application potential. Recently, the modulation of the photoluminescence emission of lanthanide-doped ferroelectric perovskites by applying an electric field has been reported. Herein, we show that the ferroelectric order and, more generally the polar order, has a direct effect on the photoluminescence of Eu^3+^ in the model BaZr_x_Ti_1−x_O_3_ perovskite even in the absence of an external field. The dipole arrangement evolves with increasing *x* from long-range ferroelectric order to short-range order typical of relaxors until the non-polar paraelectric BaZrO_3_ is achieved. The cooperative polar interactions existing in the lattice (*x* < 1) promote the off-center displacement of the Eu^3+^ ion determining a change of the lanthanide site symmetry and, consequently, an abrupt variation of the photoluminescence emission with temperature. Each type of polar order is characterized by a distinct photoluminescence behaviour.

## Introduction

The ability to tune and enhance the properties of photoluminescent materials (phosphors) by changing the emission wavelengths, intensity, band-shape, emission quantum yields and excited states lifetime is essential for optimizing the performance of devices and understanding the luminescence mechanisms. A common type of highly efficient luminescent phosphors are rare-earth (RE) doped oxides, such as Eu^3+^:Y_2_O_3_, Nd^3+^:Y_3_Al_5_O_12_ and Er^3+^:glass^1–3^. For a given host structure, the photoluminescence (PL) and, in particular the color and brightness of the emitted light, can be tailored by changing the nature and the concentration of the RE ion as well as introducing a co-dopant as sensitizer. The incorporation of the same RE in different hosts also produces modifications of PL properties due to the variation of the crystal symmetry and crystal field around the active ion. More recently, efforts have been focused on the active, *in-situ* control of the emission of luminescent materials as this would represent a real breakthrough in the phosphor technology. Real-time modulation of PL can be realized by altering the local symmetry and the energy levels by the application of an external physical stimulus or field. The strong coupling existing in ferroelectrics between the lattice strain and external variables, such as electric field and mechanical stress, offers a unique opportunity to modify the crystal field around the active ions thus realizing an effective real-time PL tuning, as demonstrated in some recent papers for RE-doped ABO_3_ ferroelectric perovskites and LiNbO_3_^4–9^. Although possible correlations between the PL response and parameters such as the remnant polarization and the poling field have been proposed and the occurrence of a field-induced phase transition has been claimed to play an important role^8,10–15^, the detailed mechanisms which control the luminescence in ferroelectric materials are not fully understood yet.

In the present paper we report strong evidence that the ferroelectric and, more generally, the polar order can control the luminescence properties of Eu^3+^ ions even without the application of an external field or stimulus and we propose a possible mechanism for explaining our observations.

The BaZr_x_Ti_1−x_O_3_ (BZT) perovskite solid solution has been selected as a model host because the polar order can be tuned by changing *x*. The end-member BaTiO_3_ (*x* = 0) is one of the most carefully investigated ferroelectric compounds and, thanks to its high dielectric constant and low losses, is widely used as a ceramic material for the fabrication of multilayer ceramic capacitors in the microelectronic industry and other applications^16,17^. BaTiO_3_ exists in different crystallographic variants, depending on temperature^18^. At the Curie temperature, *T*_*C*_ = 125–130 °C, it turns from the high temperature paraelectric cubic form (C, *Pm-3m*) to the ferroelectric tetragonal structure (T, *P4mm*). On further cooling, two other ferroelectric phases are observed: orthorhombic (O, *Amm2*), below 5–15 °C, and rhombohedral (R, *R3m*), below −80 –−90 °C. All polar forms originate from a small deformation of the prototype cubic lattice arising from the off-center displacement of the Ti ion (about 0.1 Å at room temperature) in the TiO_6_ octahedron. The cooperative, long-range ordering of the resulting electrical dipoles gives rise to macroscopic spontaneous polarization, spontaneous lattice strain and ferroelectricity^19^. Differently from BaTiO_3_, the crystal structure of the end-member BaZrO_3_ (*x* = 1) corresponds to the prototype *Pm-3m* cubic perovskite up to the melting point^20,21^. Barium zirconate is a nonpolar, paraelectric solid which has attracted attention in recent years as an efficient proton conductor when doped with acceptor impurities^22,23^. BaTiO_3_ and BaZrO_3_ show complete solid solubility. In the BZT solid solution the extent of polar order decreases with increasing *x*^24–26^ as Zr^4+^ (0.720 Å) is bigger than Ti^4+^ (0.605 Å) and does not move off the center of the BO_6_ octahedron. Long-range order typical of conventional ferroelectrics prevails for 0.0 ≤ *x* ≤ 0.15, whereas the correlation length of polar order is reduced for 0.15 < *x* ≤ 0.25 corresponding to a diffuse ferroelectric to paraelectric transition. Relaxor or dipolar glass state with only short-range polar order is observed for higher values of *x*, up to 0.95, and finally weak paraelectric behavior dominates in BaZrO_3_. The relaxor state is characterized by the presence of polar nanoregions (PNRs) embedded in a paraelectric matrix and an average cubic structure^27^. According to recent investigations^24,25^, the PNRs in BZT essentially correspond to frozen BaTiO_3_ clusters with Ti off-centering.

Eu^3+^ was chosen as a dopant because of the well-known bright emissions and for its powerful ability to act as a spectroscopic local probe for the determination of the symmetry of the first coordination sphere of a lanthanide ion in organometallic molecules and crystals^28^. Furthermore, Eu^3+^ has an ionic radius (c.n. 12) of 1.226 Å and, therefore, its crystallo chemical behavior can be considered as representative of other trivalent lanthanide ions with intermediate size, from Sm^3+^ to Er^3+^.

Six Eu-doped Ba(Ti, Zr)O_3_ ceramics with composition Ba_1−y_Eu_y_Ti_1−x−y/4_Zr_x_O_3_ (*y* = 0.01; *x* = 0, 0.05, 0.15, 0.30, 0.50, 0.70) and Ba_1−y_Eu_y_Zr_1−y/4_O_3_ (*y* = 0.01) were investigated. The different samples are labelled as BZX, where X = 100*x*.

## Results and Discussion

### Dielectric properties and polar order

**Figure 1:**
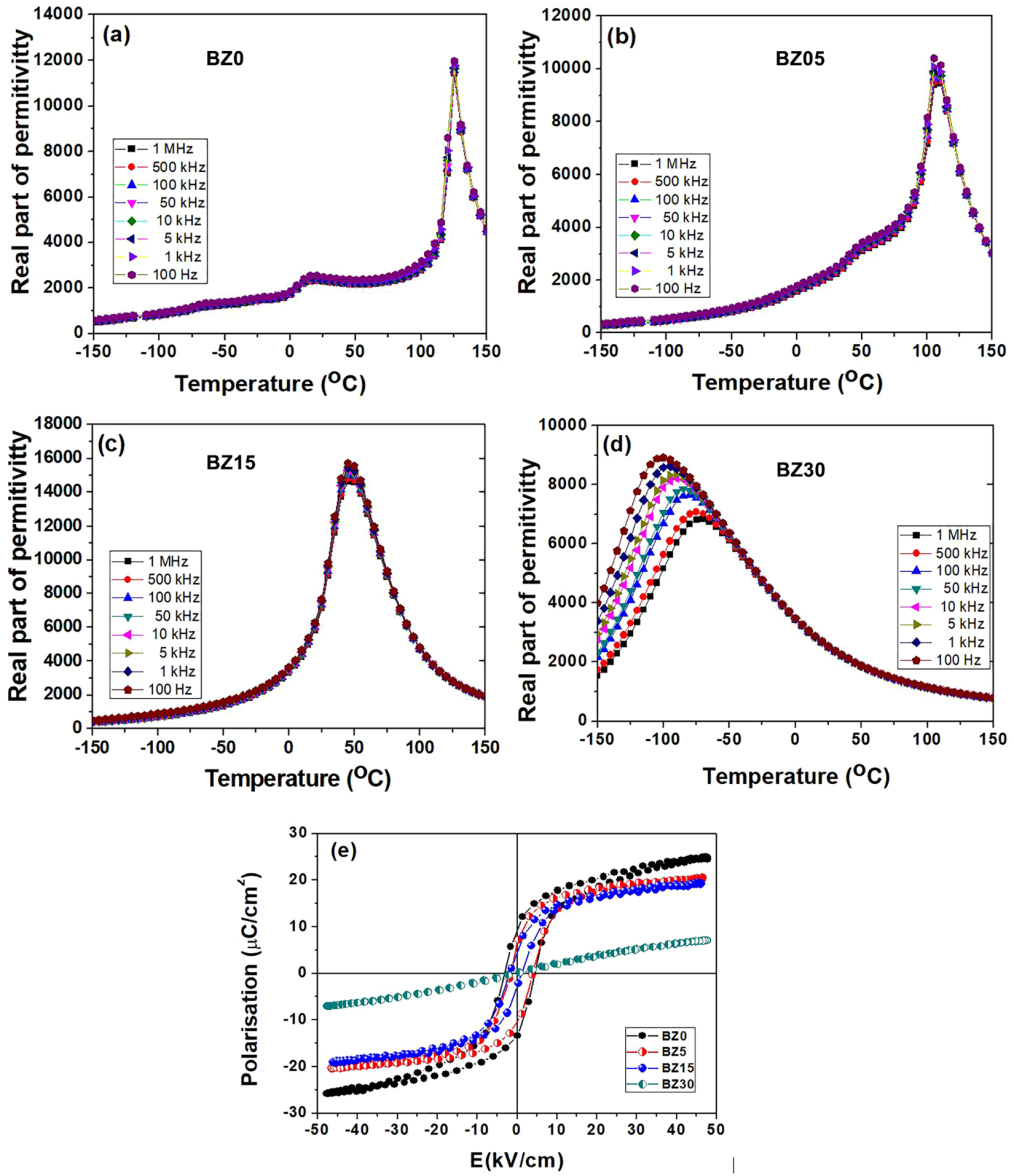
**(a–d)** Real part of permittivity (relative dielectric constant) of Ba_1−y_Eu_y_Ti_1−x−y/4_Zr_x_O_3_ (*y* = 0.01, *x* = 0.0, 0.05, 0.15 and 0.30) ceramics at 10^2^–10^6^ Hz. **(e)** Ferroelectric hysteresis loops at room temperature for different samples.

The dielectric constant (real part of permittivity) of the BZ0-BZ30 ceramics is reported in Fig. 1. The phase transitions are indicated by anomalies of the dielectric constant. When *x* = 0 (Fig. 1a), there are three peaks at −70, 15 and 125 °C corresponding to R/O, O/T and T/C transitions, respectively.

The sharp permittivity peak at *T*_*C*_ is typical of conventional ferroelectrics with first-order ferro/para transition. For ceramic BZ5 (Fig. 1b), *T*_*C*_ decreases to 108 °C and the temperature of the O/T transition increases to 50 °C.

The R/O transition is no longer detected in Fig. 1b but can still be identified from the anomaly of the dielectric loss (the ratio of the imaginary and real part of dielectric permittivity, see Figure S1, Supporting Information) at about −10 °C. A single and broad permittivity peak at 50 °C, corresponding to a diffuse R/C phase transition, is observed in Fig. 1c when *x* = 0.15. Accordingly, the dielectric loss also reveals a single transition (Figure S1, Supporting Information). The sample BZ30 (Fig. 1d) displays a typical relaxor behavior: the temperature corresponding to the maximum permittivity shifts at higher temperatures with increasing frequency. The permittivity maximum no longer corresponds to a phase transition but is determined by the relaxation dynamics of the PNRs^27^. Comparison with literature data^29–31^ indicates that the dielectric properties of the Eu-doped samples are rather similar to those reported for BZT materials likely due to the low concentration of the dopant. The phase transition temperatures of ferroelectric samples are very close (within a few degrees) to those of the undoped ceramics.

The polarization - electric field (E) loops reported in Fig. 1e confirm the results of dielectric measurements. BZ0, BZ5 and BZ15 show typical hysteretic ferroelectric behavior. Both the maximum polarization and the remnant polarization (intercept with the vertical axis) decrease with increasing *x* as expected from the progressive lowering of *T*_*C*_. BZ30 sample shows a non-linear behavior without hysteresis, a feature representative of relaxors.

### Crystal structure

The splitting of the (200) peak in the XRD pattern of the BZ0 ceramic (Figure S2, Supporting Information) indicates a typical *P4/mm* tetragonal structure and the refinement gives a c/a ratio of the lattice parameters of 1.0096, only slightly smaller than that of undoped BaTiO_3_ (1.0109). The unit cell volume, 64.35 Å^3^, is virtually the same of undoped barium titanate (64.34 Å^3^) being the difference within the experimental error. Although peak splitting is no longer observed, the broadening of some lines (compare the 111 and 200 peaks) indicates a non-cubic structure for BZ5 (Figure S2, Supporting Information), in agreement with the existence of long-range ferroelectric order at room temperature. The assignment of the crystal symmetry is not straightforward given the absence of splitting. Refinement with *P4mm* tetragonal structure with a lower c/a ratio of 1.0041 results in a marginally better fit than orthorhombic *Pbnm* structure. XRD pattern of BZ15 indicates a cubic *Pm-3m* symmetry (Figure S2) though this composition is expected to be ferroelectric at room temperature as indicated by the dielectric measurements (Fig. 1c). This apparent discrepancy is determined by the proximity of the phase transition (*T*_*C*_ = 50 °C) and, consequently, the small deviation of the polar structure from the cubic arrangement not detectable with a conventional diffractometer. The ceramics with *x* = 0.30, 0.50 and 0.70 show the cubic *Pm-3m* structure, as expected from their relaxor nature. Relaxors possess an average cubic structure but their local symmetry is lower and characterized by the existence of PNRs with short-range polar order. The unit cell volume increases linearly with *x* (Figure S2, Supporting Information) as a result of the larger ionic radius of Zr^4+^ (0.720 Å, c.n. 6) in comparison to Ti^4+^ (0.605 Å, c.n. 6). The unit cell volume of the ceramic with x = 1 is 73.71 Å^3^ to be compared with 73.83 Å^3^ of undoped BaZrO_3_.

### Photoluminescence properties

**Figure 2:**
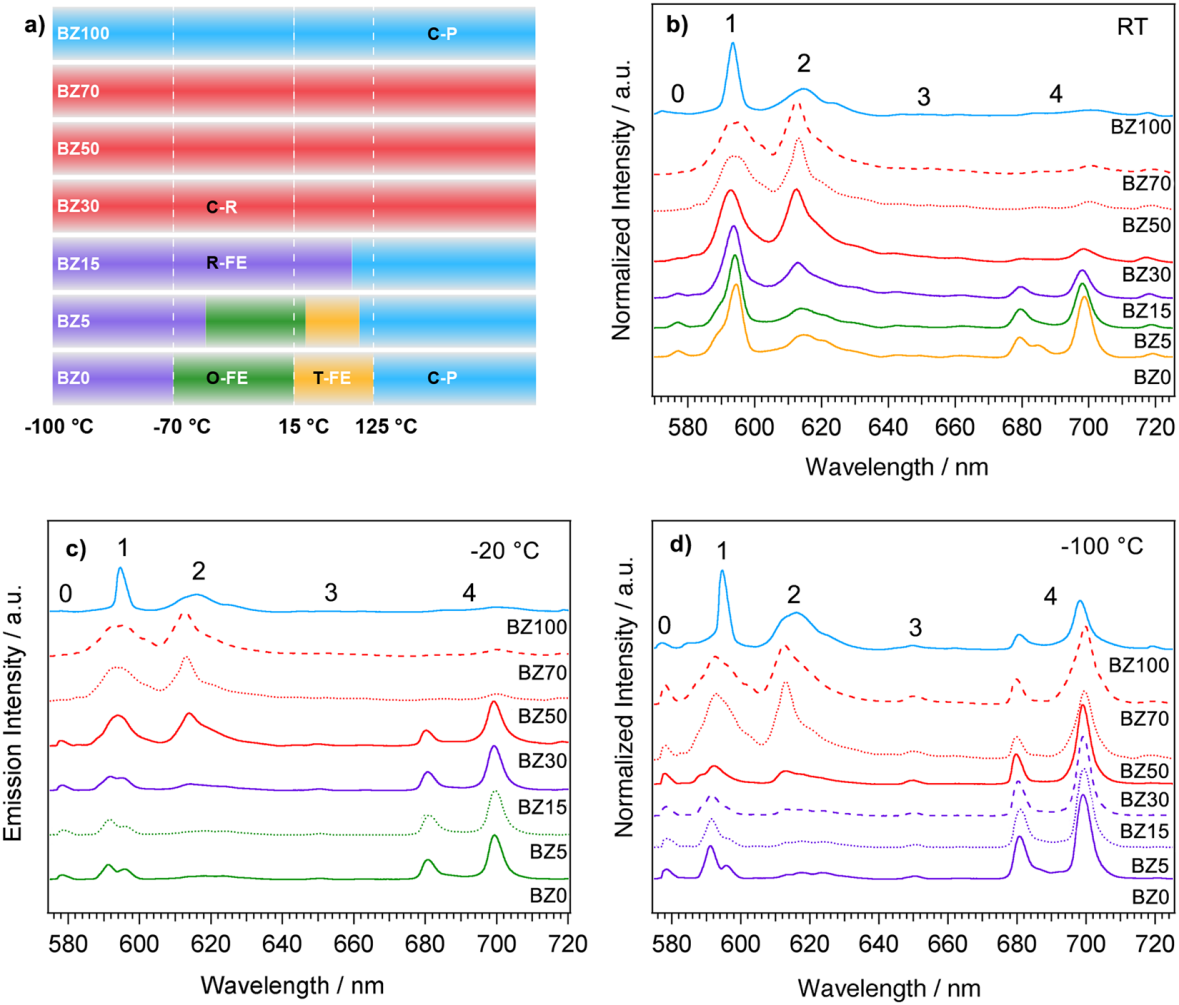
(**a**) schematic representation of the stability range of the different crystal phases or polar variants in the Ba_1−y_Eu_y_Zr_x_Ti_1−x−y/4_O_3_ and Ba_1−y_Eu_y_Zr_1−y/4_O_3_ (*y* = 0.01; *x* = 0, 0.05, 0.15, 0.30, 0.50, 0.70) ceramics. The drawings serve only as a quick eye guide to correlate structure and polar order with luminescence properties. Labels: C-P = cubic-paraelectric; C-R = cubic-relaxor; R-FE, O-FE, and T-FE = ferroelectric materials having rhombohedral, orthorhombic or tetragonal structure, respectively. Photoluminescence spectra of Eu:BZT ceramics at (**b**) room temperature, (**c**) −20 °C and (**d**) −100 °C. The curves color in panels (b-d) denotes the crystal phase and polar order according to panel (a). The numbers in panels b), c) and d) label the ^5^D_0_ → ^7^F_J_ (J = 0, 1, 2, 3, 4) Eu^3+^ transitions.

PL spectra of the investigated ceramics are reported in Fig. 2. Panel a) displays the stability range of crystal phases and polar variants as determined from the study of the dielectric properties. The drawings serve as a quick eye guide to correlate structure and polar order with luminescence properties.

The shape of room temperature emission spectra (Fig. 2b), obtained exciting the ^5^L_6_ ← ^7^F_0_ transition at 395 nm, strongly depends on sample composition. The PL spectrum of BZ0 shows four groups of bands that are characteristics for europium emission from the ^5^D_0_ excited state to the ^7^F_J_ (J = 0, 1, 2, 3, 4) manifolds located at: 578 nm (^7^F_0_), 590–596 nm (^7^F_1_), 613–623 nm (^7^F_2_) and 680–720 nm (^7^F_4_)^28^. The ^5^D_0_ → ^7^F_3_ band, if any, is quite faint. HR spectra of the ^5^D_0_ → ^7^F_1_ (Figure S3) band evidence four components indicating the presence of at least two nonequivalent Eu^3+^ centers. In fact, considering the degeneration of the J = 1 level, a maximum of three components can be observed for a single site.

To verify the absence in the emission spectra of contributions coming from interface/foreign sites, sample BZ0 was annealed at 1450 °C for 48 h. This prolonged thermal treatment determined the exaggerated growth of some grains up to a size of 300–400 μm, thus allowing the intrinsic luminescence of Eu^3+^ in the perovskite lattice to be separated from other extrinsic contributions, such as the segregation of secondary phases (e.g. Eu_2_Ti_2_O_7_) and/or glassy phase films at grain boundaries, if any. Exaggerated grain growth during prolonged sintering is quite common in pure and slightly doped BaTiO_3_ ceramics and related to the formation of a liquid phase due to local stoichiometry variations or localized impurities^32–34^.

PL spectra collected by means of a microscope on a polished cross-section at the center of a large grain and on the fine-grained matrix (Figure S4) are virtually identical. Therefore, the non-equivalent Eu^3+^ sites are associated with the perovskite lattice. Although the ionic radius of Eu^3+^ in octahedral coordination (0.947 Å) is rather large and the stoichiometry was designed for exclusive Ba-site substitution, a small amount of the rare-earth could be incorporated at the B site. The probability of Eu^3+^ incorporation in perovskite B-site increases with increasing Zr content, as the unit cell volume expands of about 15% moving from BaTiO_3_ to BaZrO_3_. Alternative and complementary explanations for the existence of multiple sites will be discussed further on studying the emission spectra as a function of temperature.

As *x* increases from 0 to 0.7, the intensity of the ^5^D_0_ → ^7^F_4_ band decreases, the ^5^D_0_ → ^7^F_2_ emission gets stronger and overcomes the intensity of the ^5^D_0_ → ^7^F_1_ transition in BZ50 and BZ70. The ^5^D_0_ → ^7^F_2_ emission is strongly reduced in sample with composition *x* = 1, and the spectrum is dominated by the sharp ^5^D_0_ → ^7^F_1_ transition. Noteworthy, a significant band broadening is observed for BZ30, BZ50 and BZ70 ceramics. These shapes closely resemble those of Eu^3+^ doped glasses in which Eu^3+^ ions occupy multiple sites having small differences in the coordination environment^35^. Indeed, for intermediate compositions a significant contribution to PL is given by several sites corresponding to a different number of ZrO_6_ (TiO_6_) octahedra surrounding the Eu^3+^ ion. Moreover, the heterogeneous local structure of the relaxor ceramics, corresponding to Ti-rich PNRs embedded in a cubic paraelectric matrix^26,27^, further increases the disorder of the europium sites.

The narrowing of the ^5^D_0_ → ^7^F_1_ line and the drop of the intensity of ^5^D_0_ → ^7^F_2_ transition observed in the emission spectra of BZ100 suggests a decrease of the number of spectroscopic sites determined by the simpler composition and the disappearance of the PNRs. Very similar spectra are reported for BaZrO_3_ and BaSnO_3_ (cubic *Pm-3m*) in which Eu^3+^ is incorporated at the Ba site^36–39^. Indeed, the ^5^D_0_ → ^7^F_1_ transition is the most intense when Eu^3+^ occupies a centrosymmetric position such as *O*_*h*_ site of Ba in BaZrO_3_ for which all transitions but ^5^D_0_ → ^7^F_1_ are forbidden.

Not only the composition but also the temperature has a strong influence on the Eu^3+^ emission. At −20 °C (Fig. 2b) the spectra of the ferroelectric ceramics (BZ0, BZ5 and BZ15) are dominated by the ^5^D_0_ → ^7^F_4_ bands whose integrated intensity is three and a half times larger compared to the ^5^D_0_ → ^7^F_1_ transition that is the most intense at room temperature. All other bands, and in particular the ^5^D_0_ → ^7^F_2_ one, have a much lower intensity. The ^5^D_0_ → ^7^F_4_ transition is the strongest even in sample BZ30 in comparison to RT. The spectra of BZ50, BZ70 and BZ100 are instead very similar to those observed at room temperature. At −100 °C (Fig. 2c), the ^5^D_0_ → ^7^F_4_ transition is the most intense for all ceramics except BZ100, for which it has the same order of magnitude as ^5^D_0_ → ^7^F_1_ and ^5^D_0_ → ^7^F_2_. No significant changes in the shape of PL spectra of ferroelectric ceramics are observed between −20 and −100 °C. It is worth noting that band broadening in the PL spectra of relaxors is unaffected by temperature variations.

**Figure 3:**
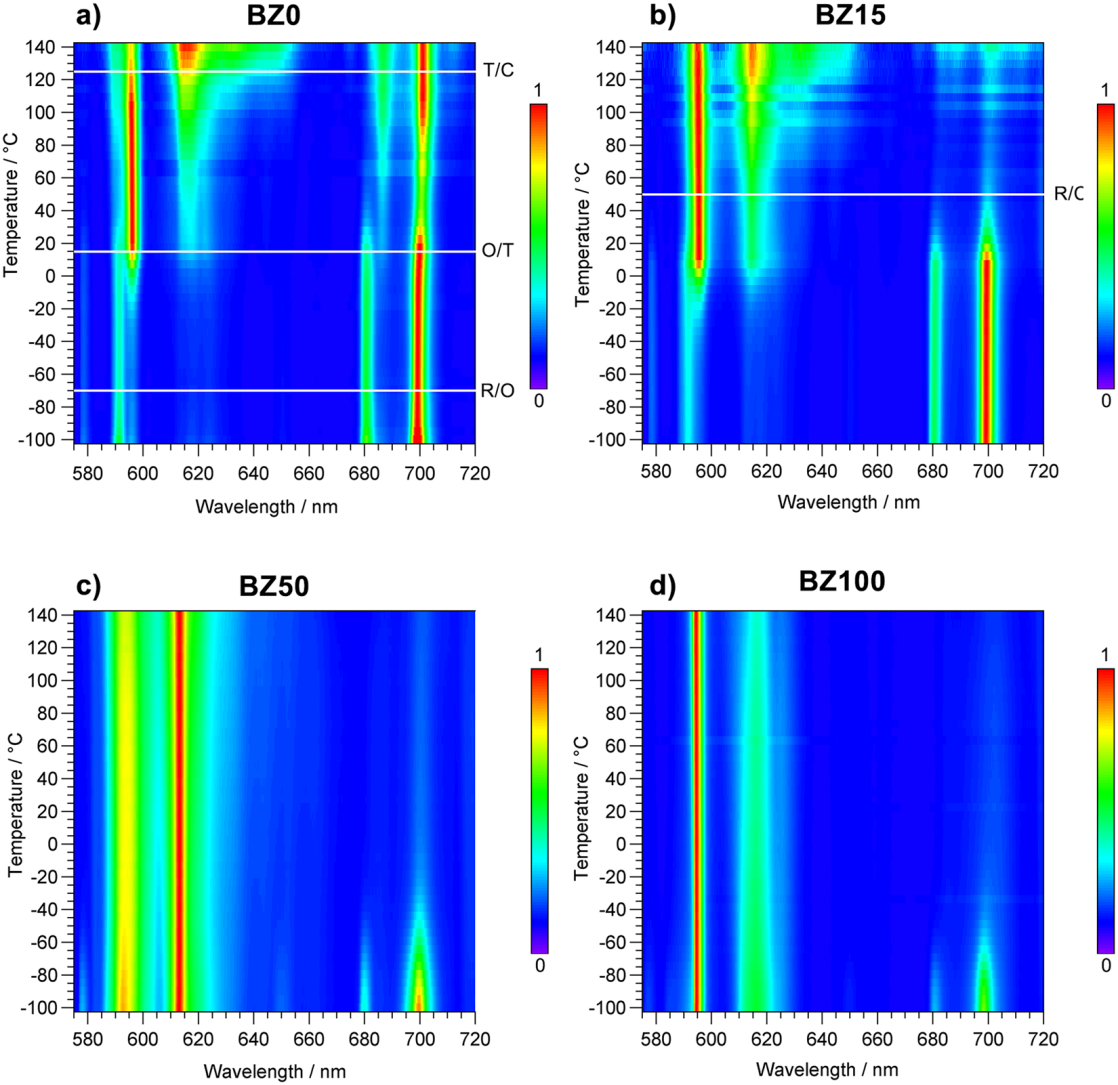
PL vs T maps of Ba_1−y_Eu_y_Zr_x_Ti_1−x−y/4_O_3_ and Ba_1−y_Eu_y_Zr_1−y/4_O_3_ (*y* = 0.01; *x* = 0, 0.15, 0.50) ceramics as a function of temperature (−100 to 140 °C). (**a)** BZ0, (**b)** BZ15, (**c)** BZ50 and (**d)** BZ100. The horizontal white lines in panels **a)** and **b)** indicate the crystal phase transition temperatures.

The detailed evolution of the PL spectra for some representative samples (BZ0, BZ15, BZ50 and BZ100) over the whole investigated temperature range, −100 to 140 °C, is illustrated in the color maps reported in Fig. 3. The maps of BZ5, BZ30 and BZ70 samples are shown in Figure S5.

Among other features, in ferroelectric Ti-rich ceramics (BZ0, BZ5 and BZ15) the main temperature-induced spectral modification is represented by the inversion of the intensity of the ^5^D_0_ → ^7^F_1_ and ^5^D_0_ → ^7^F_4_ transitions at about 10 °C, irrespective of composition. Moreover, in BaTiO_3_ the ^5^D_0_ → ^7^F_4_ transition remains strong up to 140 °C. Conversely, for relaxors we observed the rapid increase of the intensity of ^5^D_0_ → ^7^F_4_ transition, at temperatures that progressively decrease upon increasing Zr^4+^ content (*x* ≥ 0.30), and hence decreasing the number and/or the size of the Ti-rich PNRs.

It is worth noting that the spectrum of BZ100 is dominated by the ^5^D_0_ → ^7^F_1_ transition in the whole temperature range except at −100 °C. The PL variations are not determined by crystallographic modifications, as there is no correlation with the phase transitions (evidenced by white horizontal lines in Figs 3 and S5) in the ferroelectric samples while no transitions occur in relaxors.

**Figure 4:**
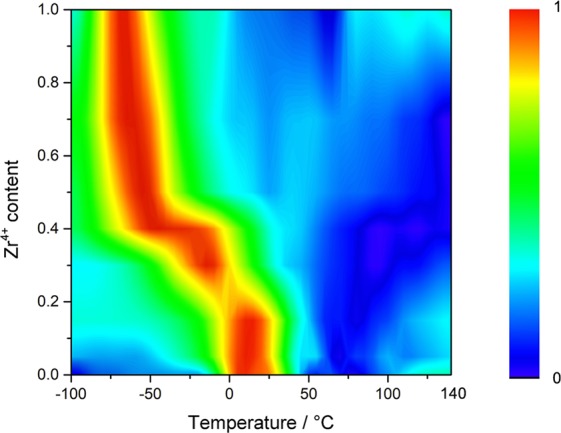
Dependence on temperature and composition of the first temperature derivative D_14_ of the ^5^D_0_ → ^7^F_1_/^5^D_0_ → ^7^F_4_ integrated intensities ratio of Ba_1−y_Eu_y_Zr_x_Ti_1−x−y/4_O_3_ and Ba_1−y_Eu_y_Zr_1−y/4_O_3_ (*y* = 0.01; *x* = 0, 0.05, 0.15, 0.30, 0.50, 0.70) ceramics. The color scale refers to the D_14_ normalized values.

It is evident that the emission properties of the solid solutions are mainly affected by the nature of polar order: ferroelectric, relaxor or paraelectric. The evolution of the PL spectra with temperature and polar order can be better quantified by plotting the first temperature derivative (D_14_) of the ^5^D_0_ → ^7^F_1_/^5^D_0_ → ^7^F_4_ integrated intensities ratio (Figs 4 and S6).

The temperature at which *D*_14_ reaches its maximum value is sensitive to the polar order of BaZr_x_Ti_1−x_O_3_. For ferroelectric ceramics (BZ0, BZ5 and BZ15), the maximum of *D*_14_ is at ca. 10 °C and does not change with composition. By contrast, it progressively shifts to lower temperatures in relaxor and paraelectric ceramics on increasing Zr^4+^ content: −13 °C for BZ30, −55 °C for BZ50, −60 °C for BZ70, −65 °C for BZ100.

The absence of correlation between crystal phase transitions and modifications of PL emission with temperature is not surprising. The local symmetry of the Eu^3+^ site and, consequently, the relative intensities and shape of ^5^D_0_ → ^7^F_J_ manifolds in europium emission spectra are indeed essentially determined by the geometry of the first and second coordination sphere of Eu^3+^ rather than by the long-range crystallographic structure. There is increasing evidence that the local structure of BaTiO_3_ remains rhombohedral at all temperatures as indicated by the results of EXAFS, NMR and the pair-distribution function investigations^40–45^. According to the largely accepted eight-site model^46^, the Ti ions in the paraelectric cubic phase exhibit off-center local displacement along the eight [111] directions but the average polarization is null because of the random distribution among the 8 split sites. Below the Curie temperature partial ordering occurs. Thus O, T and C long-range structures are the result of the averaging [111] displacements of octahedral Ti over 2, 4 and 8 local rhombohedral directions, respectively. Only for the rhombohedral phase the local and long-range structures are equivalent. It is worth noting that the deformation of the R phase with respect to the cubic unit cell is quite small. At −93 °C the rhombohedral angle α is 89.85°^18^, *i.e*. there is only a deviation of 0.15° from the cubic prototype perovskite. Noteworthy, the position of Ba^2+^ ions is not affected by the off-center displacement of Ti^4+^ ions.

According to the above results (see Figs 2, 3 and S6), the ferroelectric ceramics show PL emission dominated by the ^5^D_0_ → ^7^F_4_ transitions over a broad temperature range, from −100 °C up to room temperature or even higher (up to 60–80 °C for *x* = 0). Emission spectra characterized by an abnormally high intensity of ^5^D_0_ → ^7^F_4_ transition are reported for Eu^3+^ occupying *D*_*4d*_ (or distorted *D*_*4d*_) sites with coordination geometry close to square antiprism^28,47–50^ as well as distorted *D*_*2d*_ sites^51^. For an undistorted *D*_*4d*_ symmetry the ^5^D_0_ → ^7^F_2_ transition is forbidden whereas the transitions ^5^D_0_ → ^7^F_1_ and ^5^D_0_ → ^7^F_4_ are allowed^28^. This situation is uncommon and occurs only for the point groups *D*_*4d*_ and *O*.

The ionic radius (c.n. 12) of Eu^3+^ (1.226 Å) is much smaller than that of Ba^2+^ (1.610 Å) and, consequently, the lanthanide ion is over-coordinated when it is positioned at the Ba site and the possibility of occupying a lower symmetry site has to be considered. Very recent investigations including careful crystal structure determination from synchrotron X-ray diffraction data and first-principle calculations on Gd- and Dy-doped BaTiO_3_^52,53^ as well as the study of dielectric relaxations in Gd- and Dy-doped SrTiO_3_^54,55^ have provided some evidence about the off-center displacement of trivalent lanthanide ions with average size at A-site of these perovskites.

**Figure 5:**
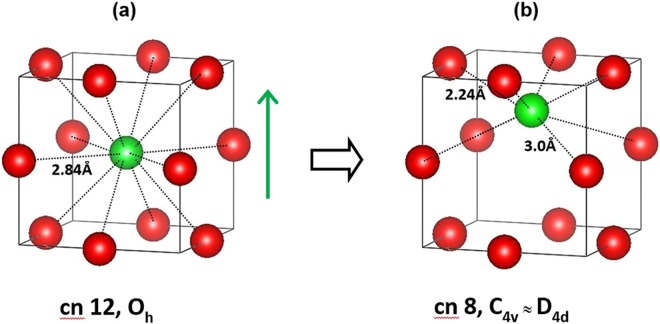
Evolution of local symmetry during the off-center displacement of Eu^3+^ along one of the [100] directions of cubic BaTiO_3_. See text for details. Red spheres: O^2−^. Green sphere Eu^3+^. The images were realized using VESTA^56^.

As illustrated in Fig. 5, and referring to the cubic structure for the sake of simplicity, a displacement of about 1.0 Å along one of the six [100] directions (towards the center of any of the cube faces) drives Eu^3+^ from the regular dodecahedral Ba site, with *O*_*h*_ symmetry, to an acentric *C*_*4v*_ site, with approximate *D*_*4d*_ symmetry corresponding to a distorted 8-coordinated square antiprism.

The distortion arises from the existence of two groups of Eu-O distances: 2.240 and 3.000 Å (the first value is similar to the Eu-O distance in Eu_2_O_3_ and Eu_2_Ti_2_O_7_, 2.2–2.3 Å). In principle, the lanthanide can also move in other directions. Off-center displacement of Eu^3+^ along the crystallographic directions [110] (i.e. along a straight line parallel to the diagonal of the cube face) and [111] (towards a corner of the unit cell) will result in a reduction of the coordination number to 7 and 9, respectively. The local symmetry will be *C*_*2v*_ for displacement in the [110] direction and *C*_*3v*_ for displacement in the [111] direction.

When Eu^3+^ ions occupy sites with this punctual symmetry, the ^5^D_0_ → ^7^F_0_ transition is allowed and can be observed in the emission spectra. In our case, at room temperature the ^5^D_0_ → ^7^F_0_ transition is present only in PL spectra of ferroelectric materials and between −20 °C and −100 °C in relaxors depending on their composition, indicating a non-zero probability for displacements along [110] and [111] directions.

From a purely geometric point of view, the volume (5.34 Å^3^) of the pyramidal cavity available to host Eu^3+^ when moving along the [100] and [110] directions is twice that of the space available in the [111] direction and, consequently, this latter option is less likely. It is worth noting that no displacement would be expected if Eu^3+^ were incorporated at the Ti/Zr site, as its ionic radius in octahedral coordination (0.947 Å) is exceedingly large. The geometry depicted in Fig. 5b is only a first approximation as the oxygen ions around Eu^3+^ will undergo some relaxation from their ideal positions.

According to the previous discussion, the behavior of Eu^3+^ in the two perovskites BaTiO_3_ and BaZrO_3_ is, at a first glance, counterintuitive. The unit cell volume of BaZrO_3_ (73.83 Å^3^ at room temperature) is significantly larger (+15%) compared to BaTiO_3_ (64.34 Å^3^), but in BaZrO_3_ Eu^3+^ mainly occupies the *O*_*h*_ site of Ba^2+^ from 140 °C down to at least −60 °C. The spectra are dominated by the ^5^D_0_ → ^7^F_1_ transition and no ^5^D_0_ → ^7^F_0_ and ^5^D_0_ → ^7^F_4_ emissions were detected in this temperature range for pure zirconate (see Figs 2 and 3). At variance, in Ti-rich BaZr_x_Ti_1−x_O_3_ ceramics (*x* = 0–0.15) there is convincing evidence of the displacement of Eu^3+^ along the [100] direction in the 8-fold square antiprism coordination site (Fig. 5), over a broad temperature range. Likewise, some displacements along [110] and [111] can be achieved.

The much smaller ionic radius of Eu^3+^ (1.226 Å) in comparison to Ba^2+^ (1.61 Å) is not enough by itself to explain the off-centering of the lanthanide in the ferroelectric ceramics and an additional driving force must exist. The investigation of the strikingly different behavior of Ca_x_Ba_1−x_TiO_3_ and Sr_x_Ba_1−x_TiO_3_ solid solutions provide some suggestions. While the incorporation of Sr^2+^ determines a continuous decrease of Curie temperature and unit cell volume, in the case of Ca^2+^ substitution the Curie temperature remains unaffected (stabilization of the ferroelectric phase) though the cell volume decreases. A combination of experimental techniques, first-principle calculations and atomistic modelling^57–59^ revealed the off-center displacement of Ca^2+^. Furthermore, the Ca^2+^ displacement amplifies the ferroelectric off-centering of Ti cations nearest to the Ca^2+^ site in a cooperative process thus enhancing ferroelectricity despite volume decrease. Atomistic simulations using large supercells^59^ showed that [100] displacement is preferred over [111]. Opposite, the position of Sr cations is largely central. The different behavior of the two earth-alkaline cations is essentially determined by their different ionic radius (c.n. 12), 1.34 Å for Ca^2+^ and 1.44 Å for Sr^2+^, being their chemical properties similar. It is worth noting that Ca^2+^ in CaTiO_3_ (orthorhombic *Pbnm* perovskite structure) occupies 8-coordinated distorted sites with Ca-O distances between 2.36 and 2.67 Å. The remaining 4 distances are >3 Å.

Following the same reasoning, the additional driving force determining off-centering of Eu^3+^ in ferroelectric ceramics is the enhancement of polar order and ferroelectricity resulting from the correlated movement of Eu^3+^ and Ti^4+^ ions. The off-center displacement of Eu^3+^ will give rise to an electrical dipole. If Eu^3+^ preferentially moves along one of the [100] directions in such a way that the component of the associated dipole moment will positively contribute to the dipole moment corresponding to the [111] relaxation of the Ti ions, the europium displacement will concur to the overall polarization and ferroelectricity. This mechanism can be amplified by the deformation of the TiO_6_ octahedra nearest to Eu^3+^ as happens with Ca^2+^. Opposite, if Eu^3+^ moved randomly, there would be no net contribution to ferroelectricity and no extra energy gain determined by off-centering. Hence, the absence of polar order in BaZrO_3_ prevents a correlated displacement of Eu^3+^ and Zr^4+^ and the lanthanide will largely stay in central position. It should be kept in mind that the concentration of Eu^3+^ ions is as low as 1% and, consequently, their mutual interaction will be negligible thus hampering a cooperative behavior of the lanthanide ions alone.

The evolution of the PL spectra with temperature is mainly ascribed to the change of the population of the different Eu^3+^ sites. For ferroelectric ceramics, the off-centering of Eu^3+^ occurs over a broad temperature range (up to 60–80 °C for BaTiO_3_) by the cooperative polar interactions discussed above and made possible by the existence of ferroelectric order. The polar interactions counteract the thermal motion which would favor the occupation of the central sites or more disordered distributions. In the absence of polar interactions, as in BaZrO_3_, off-centering of some Eu^3+^ can only occur at low temperature (≤−80 °C), as indicated by the increasing contribution of the ^5^D_0_ → ^7^F_4_ transition to the overall emission (Figs 2 and 3), thanks to the reduced thermal motion. In relaxors (BZ30, BZ50, BZ70), Eu^3+^ off-center displacement should be confined within PNRs, whereas a behavior similar to that exhibited by BaZrO_3_ is expected in the paraelectric matrix. However, the PL spectra of BZ30 below −40 °C are very similar to those of the ferroelectric samples (Figs 2, 3 and S5). This observation supports the idea that the ferroelectric to relaxor crossover in BaZr_x_Ti_1−x_O_3_ occurs over the composition range 0.25 ≤ x < 0.35 with coexistence of PNRs and ferroelectric domains, the latter originated by percolation of polar clusters on cooling^27,60^. The rapid increase of the ^5^D_0_ → ^7^F_4_ emission at lower temperature in the other two relaxors (Figs 2–4 and S5) is again attributed to the suppression of thermal motion and off-centering of some Eu^3+^ ions.

One can wonder if the evolution of the PL spectra with temperature might be determined by a change of defect chemistry, for example by a redistribution of Eu^3+^ ions between A and B sites in the perovskite. However, owing to the very low mobility of cation and oxygen vacancies in the investigated temperature range, the defect chemistry of BaTiO_3_ and BaZrO_3_ is essentially frozen. Oxygen vacancies, the most mobile kind of ionic defects, start to be mobile above 400 °C^61^. A change in the distribution of Eu^3+^ between Ba and Ti sites is unlikely because this would imply a complete re-equilibration of the bulk defect chemistry and in particular the concentration of the charge compensating defects (cation vacancies for Ba-site incorporation, oxygen vacancies for B-site substitution) which can only occur by long-range diffusional transport. In contrast, the change of the Eu^3+^ position from the center of the dodecahedron to a nearby acentric site occurs over a very short distance (≈1 Å) and does not require diffusion.

## Conclusions

An effective tuning of the photoluminescence of rare-earth ions incorporated in ferroelectric perovskites has recently been achieved by exploiting the strong coupling between the lattice strain and the electric field (or the mechanical stress) typical of ferroelectric materials. However, as shown in the present paper, the polar order and, in particular, the ferroelectric order, has a direct and remarkable impact on the photoluminescence of Eu^3+^ in the BaZr_x_Ti_1−x_O_3_ perovskite even in the absence of an external field.

In the ferroelectric ceramics (*x* = 0–0.15), the photoluminescence spectra display a crossover from a dominating ^5^D_0_ → ^7^F_1_ emission above room temperature to a dominating ^5^D_0_ → ^7^F_4_ emission at lower temperature. This behavior provides strong evidence of the off-center displacement of Eu^3+^ along one of the [100] directions of the lattice into a site with approximate *D*_*4d*_ symmetry with a coordination polyhedron close to a square antiprism with decreasing temperature. The off-centering of the lanthanide from the regular dodecahedral O_h_ site of Ba^2+^ can be attributed to the cooperative polar interactions with the ferroelectric lattice, *i.e*. the correlated displacement of Eu^3+^ and Ti^4+^ ions aided by the smaller ionic radius of Eu^3+^ (1.226 Å) in comparison to Ba^2+^ (1.610 Å). Conversely, in BaZrO_3_ the absence of ferroelectric order prevents a correlated displacement of Eu^3+^ and Zr^4+^ and the lanthanide will largely occupy the central dodecahedral *O*_*h*_ site in spite of the larger unit cell of the zirconate in comparison to BaTiO_3_. Only at low temperature (−100 °C) the suppression of thermal motion allows off-centering of some Eu^3+^ ions.

Materials with compositions *x* = 0.30–0.70 have a short-range polar order typical of relaxors and their photoluminescence shows a distinct crossover but a more complex behavior. The complexity partly arises from the heterogeneous nature of the relaxors at the nanoscale, corresponding to BaTiO_3_ polar nanoregions embedded in a paraelectric matrix. Moreover, for these intermediate compositions several Eu^3+^ sites corresponding to a different number of ZrO_6_ (TiO_6_) octahedra surrounding the lanthanide will give a significant contribution to the luminescence.

It is expected that other RE^3+^ ions with intermediate size (Sm^3+^ to Er^3+^), when incorporated at the Ba site of the perovskite, can exhibit a behavior similar to that of Eu^3+^ as the underlying mechanism is rather general and not restricted to a specific ion. However, the magnitude of the off-center displacement is likely to depend on the ionic radius of the dopant producing a systematic trend. Furthermore, a ferroelectric/polar order control of photoluminescence could be observed in other perovskites.

## Experimental Section

### Sample preparation

Six Eu-doped Ba(Ti,Zr)O_3_ ceramics with composition Ba_1−y_Eu_y_Ti_1−x−y/4_Zr_x_O_3_ (*y* = 0.01; *x* = 0, 0.05, 0.15, 0.30, 0.50, 0.70) and Ba_1−y_Eu_y_Zr_1−y/4_O_3_ (*y* = 0.01) were prepared by the classical solid-state route using industrial electronic grade precursors: BaCO_3_ (Solvay Bario e Derivati, Italy), TiO_2_ (Evonik Degussa grade P25, Germany), ZrO_2_ (Toho grade TZ0, Japan) and Eu_2_O_3_ (Metall Rare Earth Ltdl, China) powders as raw materials. The compositions correspond to Eu^3+^ substitution at the Ba site with Ti vacancy compensation (0 ≤ *x* ≤ 0.7) and Zr vacancy compensation (*x* = 1). The different samples are labelled as BZX, where X = 100*x*. Predominant substitution at the Ba site of BaTiO_3_ is reported for Ba/Ti < 1 up to 2–3 at.% europium,^62,63^ the solubility limit of trivalent europium. The low dopant concentration adopted (1 mol.%) guarantees that the structural and physical properties of the materials are not significantly affected. Precursor powders were wet-mixed in polypropylene jars using water as liquid and a solution of ammonium polyacrylate (pH = 10) as dispersant. After drying, the mixed powder was calcined for 4 h at 1000 °C and then compacted in cylinders (length: 1 cm, diameter: 1 cm) by isostatic pressing at 1500 bar. The resulting greens were sintered in air for 4 h at different temperatures depending on composition: 1450 °C (BZ0-BZ30), 1550 °C (BZ50-BZ70) and 1600 °C (BZ100). The relative density of the final samples was determined by the Archimedes’ method. The ceramics are well densified with relative density ≥94%. Ceramic disks with a thickness of about 1 mm were cut from the sintered body and characterized by different techniques.

### Crystal structure, dielectric and ferroelectric properties

The phase composition and crystal structure was investigated by X-ray diffraction (XRD) using a CubiX diffractometer (Panalytical, The Netherlands) with Cu Kα radiation (30 kV, 30 mA). The lattice parameters were determined using FullProf 2000. For dielectric measurements, Ag-Pd electrodes were deposited on the plane-parallel polished surfaces of the disks followed by annealing in air at 500 °C for 12 h. The dielectric properties were measured by an impedance bridge E4980A Precision LCR Meter (Agilent, Santa Clara, CA) and a dielectric spectrometer CONCEPT40 (Novocontrol Technologies, Hundsangen, Germany) in the temperature range −150–150 °C at 10^2^–10^6^ Hz. Polarization – electric field ferroelectric loops were recorded at room temperature on the electroded ceramics immersed in transformer oil bath by a Sawyer–Tower modified circuit fed by triangular high voltage wave (frequency: 10 Hz) by using a TREK amplifier.

### Photoluminescence

Room temperature luminescence spectra were recorded on solid samples in a front-face acquisition geometry with a spectrofluorimeter (Fluorolog-3, Horiba JobinYvon) equipped with double-grating monochromator in both the excitation and emission sides, coupled to a R928P Hamamatsu photomultiplier and a 450 W Xe arc lamp as the excitation source. The emission spectra were corrected for detection and optical spectral response of the spectrofluorimeter supplied by the manufacturer.

HR spectra and temperature dependent experiments (−100–140 °C) were carried out in backscattering geometry using a Horiba T64000 triple spectrometer equipped with a Peltier-cooled charge-coupled device detector (Horiba Synapse). The scattered radiation was collected through a 10× microscope objective (Olympus MPLAN, 10×/0.25). The spectrograph, equipped with 2400 lines/mm gratings for high resolution and 300 lines/mm for temperature dependent experiments, was used as a single stage spectrograph. Temperature dependent experiments were performed by means of a Linkam THMS600 heating/freezing microscope stage having temperature stability <0.1 °C over −196 °C to 600 °C temperature range.

## Supplementary information

Supplementary Information

## Data Availability

All data generated or analysed during this study are included in this published article (and its Supplementary Information files).
