# Supplementary material for: Ferroelectric order driven Eu3+ photoluminescence in BaZrxTi1−xO3 perovskite
Source: Sci Rep. 2019 Apr 23;9:6441. doi: 10.1038/s41598-019-42897-1 (PMC6478923; doi:10.1038/s41598-019-42897-1)
Supplement: Supplementary file 1 — Supplementary Information [file 41598_2019_42897_MOESM1_ESM.docx]

Ferroelectric order driven Eu^3+^ photoluminescence in BaZr_x_Ti_1-x_O_3_ perovskite

Giovanna Canu,^1^ Gregorio Bottaro,^2,*^ Maria Teresa Buscaglia,^1^ Chiara Costa,^1^ Oana Condurache,^3^ Lavinia Curecheriu,^3^ Liliana Mitoseriu,^3^ Vincenzo Buscaglia,^1,*^ Lidia Armelao^2,4^

^1^ICMATE-CNR, Via De Marini 6, 16149 Genoa, Italy

^2^ICMATE-CNR and INSTM, Department of Chemical Sciences, University of Padua, Via F. Marzolo 1, 35131 Padua, Italy

^3^Department of Physics, Alexandru Ioan Cuza University, 11 Blvd. Carol I, 700506 Iasi, Romania

^4^Department of Chemical Sciences, University of Padua, Via F. Marzolo 1, 35131 Padua, Italy

**Supplementary Information**

**FIGURE S1**

| **x = 0.0** | **x = 0.05** |
| --- | --- |
| **x = 0.15** | **x = 0.30** |

**Figure S1**. Dielectric loss of Ba_1-y_Eu_y_Zr_x_Ti_1-x-y/4_O_3_ (*y* = 0.01; *x* = 0, 0.05, 0.15, 0.30) ceramics between -150 and 150 °C at 10^2^-10^6^ Hz (see legend).

**FIGURE S2**


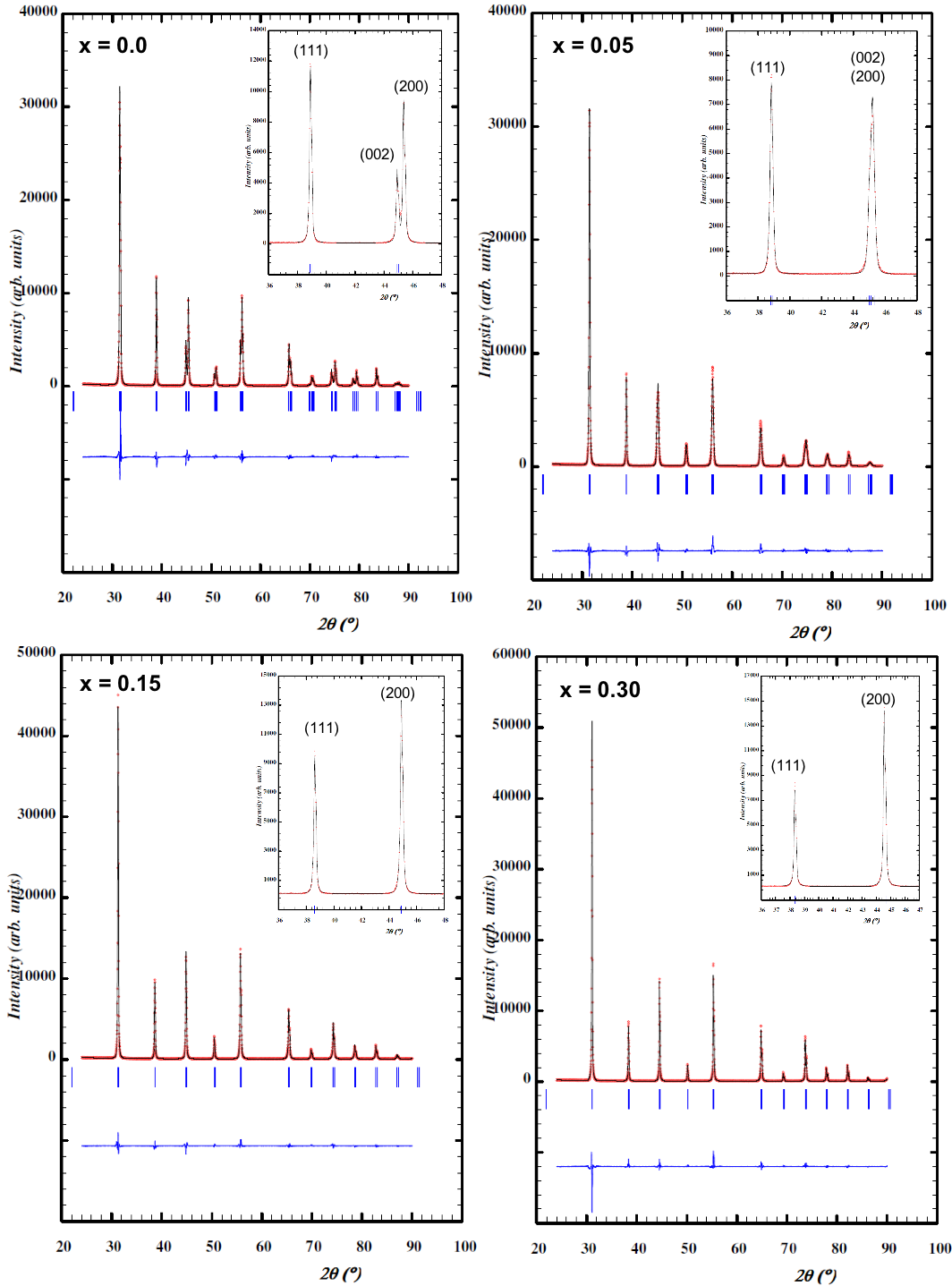

**x = 0.05**

(111)

(002)

(200)

**x = 0.0**

(002)

(200)

(111)

**x = 0.15**

(111)

(200)

**x = 0.30**

(111)

(200)


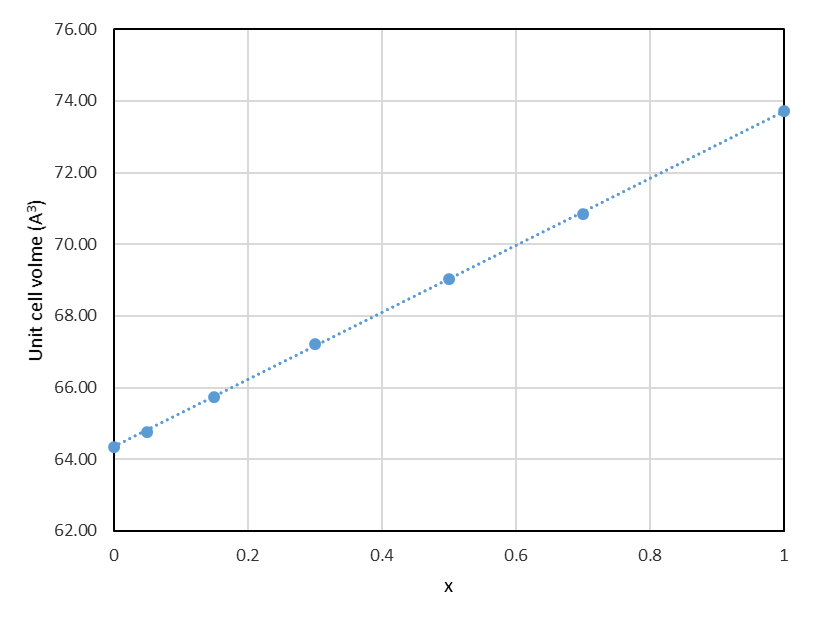


**Figure S2**. XRD patterns of Ba_1-y_Eu_y_Zr_x_Ti_1-x-y/4_O_3_ (*y* = 0.01; *x* = 0, 0.05, 0.15, 0.30) ceramics and unit cell volume *vs*. composition *x*.

**FIGURE S3**


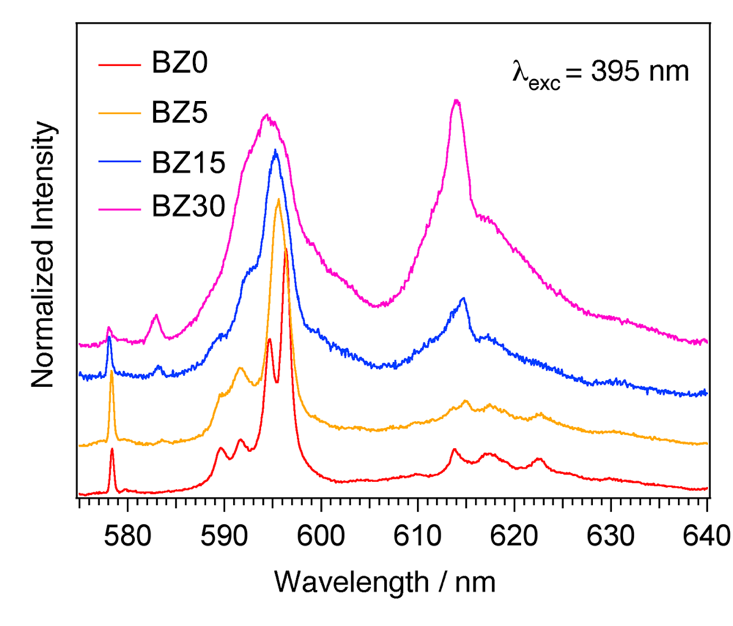


**Figure S3**. High resolution room photoluminescence spectra of BZ0, BZ5, BZ15 and BZ30 samples.

**FIGURE S4**


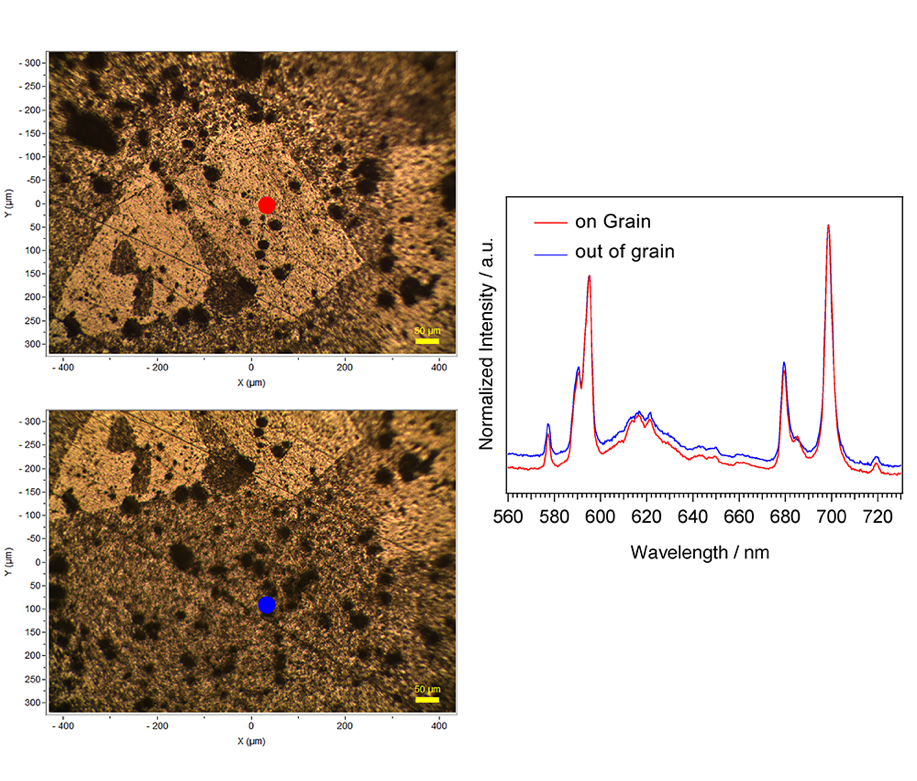


**Figure S4**. Room-temperature photoluminescence spectra of Ba_1-y_Eu_y_Zr_x_Ti_1-x-y/4_O_3_ (*y* = 0.01; *x* = 0.0, BZ0) ceramic sintered for 48 h at 1450 °C. The red spectrum was collected at the center of the large rectangular crystal in the top optical micrograph, whereas the black spectrum was collected in the polycrystalline matrix.

**FIGURE S5**


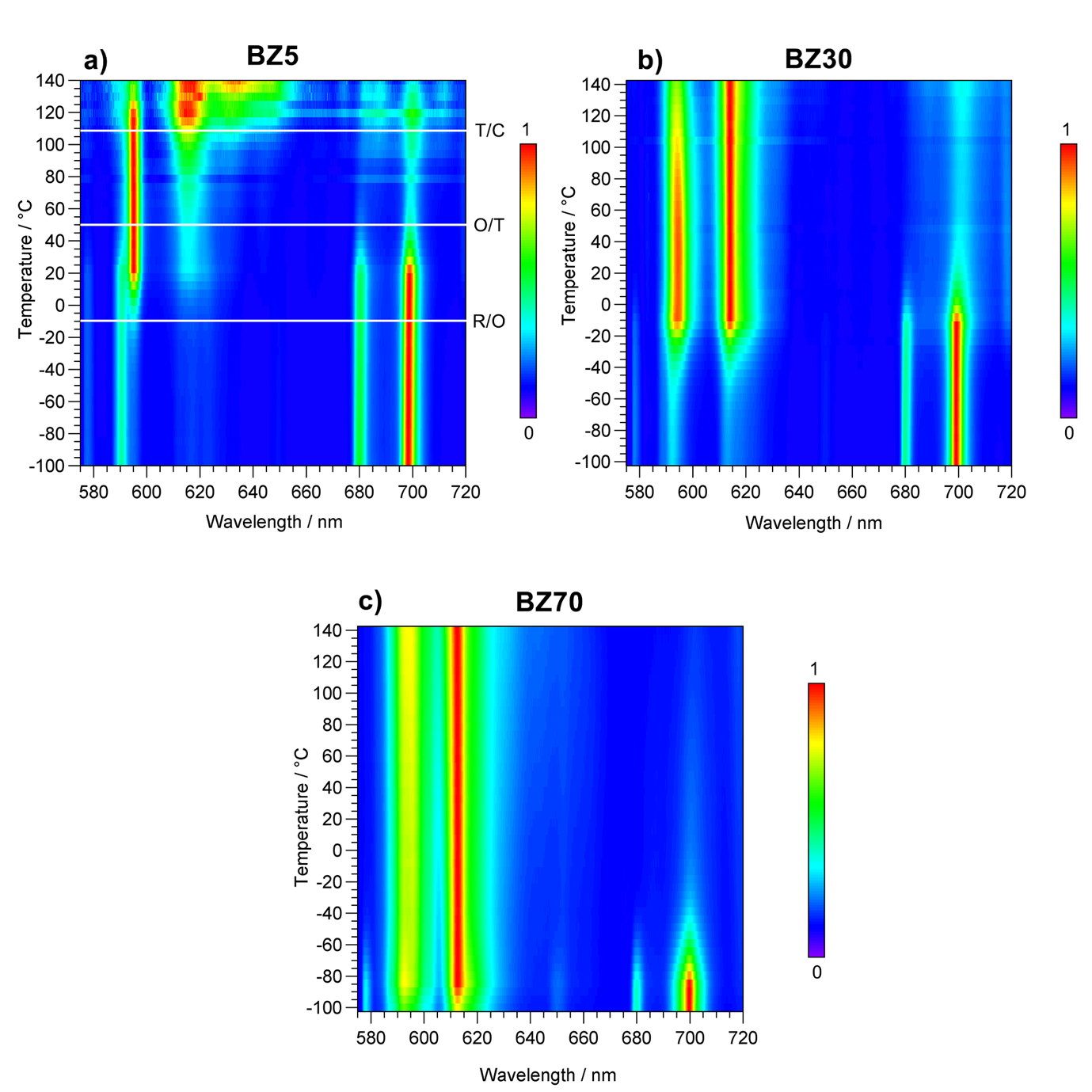


**Figure S5**. PL *vs*. T maps of Ba_1-y_Eu_y_Zr_x_Ti_1-x-y/4_O_3_ (*y* = 0.01, *x* = 0.05, 0.30 and 0.70) ceramics as a function of temperature (-100 to 140 °C). **a)** BZ5, **b)** BZ30, **c)** BZ70. The horizontal white lines in panel a) indicate the phase transition temperatures.

**FIGURE S6**


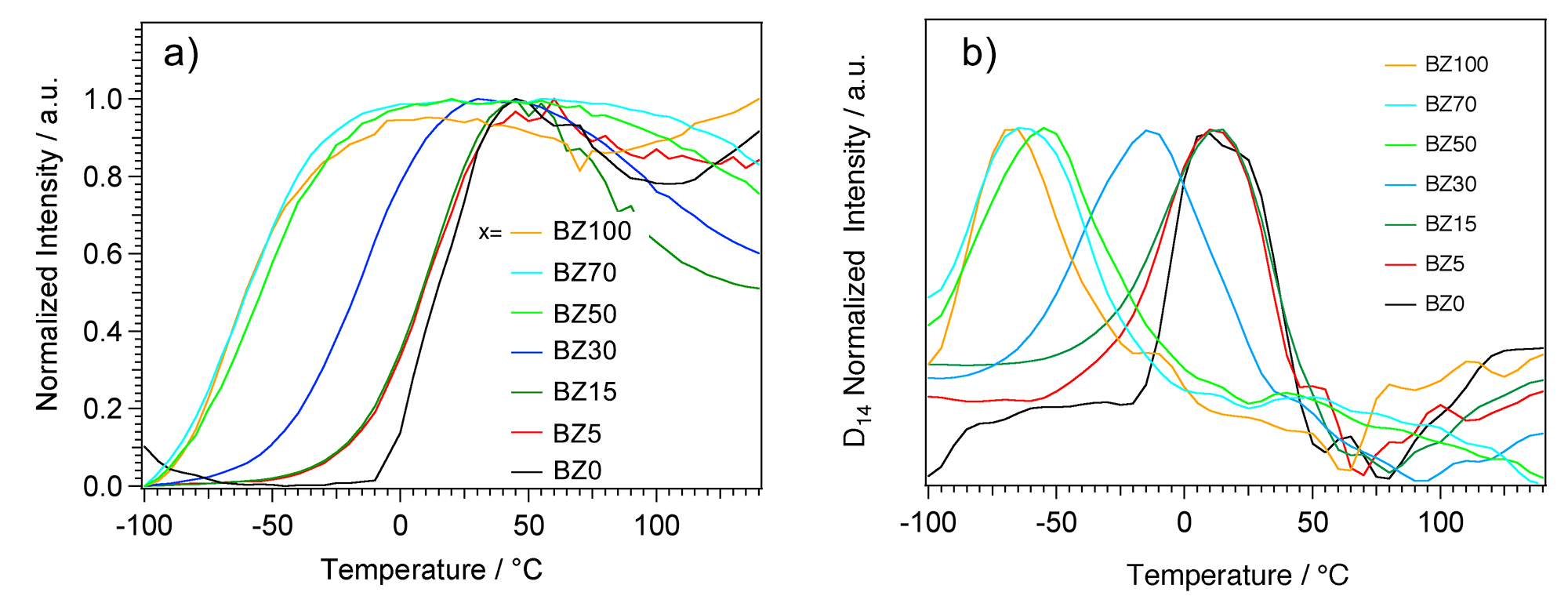


**Figure S6**. Dependence on temperature and composition of ^5^D_0_ ⭢ ^7^F_1_/^5^D_0_ ⭢ ^7^F_4_ integrated intensities ratio (**a**) and its first derivative D_14_ (**b**).
